# The landscape of targeted therapies for cholangiocarcinoma: current status and emerging targets

**DOI:** 10.18632/oncotarget.8775

**Published:** 2016-04-18

**Authors:** Dawn Q. Chong, Andrew X. Zhu

**Affiliations:** ^1^ Massachusetts General Hospital Cancer Center, Harvard Medical School, Boston, MA, USA; ^2^ Division of Medical Oncology, National Cancer Centre Singapore, Singapore

**Keywords:** cholangiocarcinoma, genetics, IDH, FGFR2

## Abstract

Cholangiocarcinoma (CCA) is a relatively rare malignancy that arises from the epithelial cells of the intrahepatic, perihilar and distal biliary tree. Intrahepatic CCA (ICC) represents the second most common primary liver cancer, after hepatocellular cancer. Two-thirds of the patients with ICC present with locally advanced or metastatic disease. Despite standard treatment with gemcitabine and cisplatin, prognosis remains dismal with a median survival of less than one year. Several biological plausibilities can account for its poor clinical outcomes. First, despite the advent of next generation and whole exome sequencing, no oncogenic addiction loops have been validated as clinically actionable targets. Second, the anatomical, pathological and molecular heterogeneity, and rarity of CCA confer an ongoing challenge of instituting adequately powered clinical trials. Last, most of the studies were not biomarker-driven, which may undermine the potential benefit of targeted therapy in distinct subpopulations carrying the unique molecular signature. Recent whole genome sequencing efforts have identified known mutations in genes such as epidermal growth factor receptor (*EGFR*), Kirsten rat sarcoma viral oncogene homolog (*KRAS*), v-raf murine sarcoma viral oncogene homolog (*BRAF*) and tumor protein p53 (*TP53*), novel mutations in isocitrate dehydrogenase (*IDH*), BRCA1-Associated Protein 1 (*BAP1*) and AT-rich interactive domain-containing protein 1A (*ARID1A*), and novel fusions such as fibroblast growth factor receptor 2 (*FGFR2*) and ROS proto-oncogene 1 (*ROS1*). In this review, we will discuss the evolving genetic landscape of CCA, with an in depth focus on novel fusions (e.g. *FGFR2* and *ROS1*) and somatic mutations (e.g. *IDH1/2*), which are promising actionable molecular targets.

## INTRODUCTION

Cholangiocarcinoma (CCA) comprises of malignancy arising from the intrahepatic, perihilar and distal biliary tree. Intrahepatic CCA (ICC) is the second most common primary hepatic malignancy, after hepatocellular carcinoma, and accounts for 10-20% of primary liver cancers [1, 2]. The incidence and mortality rates of ICC have been rising worldwide in the past decade, whereas those of extrahepatic CCA (ECC) are either stable or decreasing [2]. In the Western countries, the annual incidence of ICC is 2.1 per 100,000 person years [3]. Chronic inflammation from liver fluke infestation, hepatitis B and C infections, primary sclerosing cholangitis and inflammatory bowel disease are the main risk factors of CCA [4]. Other less common etiologic factors include hepatolithiasis, cirrhosis, alcohol, smoking, fatty liver disease and cholelithiasis [1].

Only 10-15% of the patients with CCA are amenable to potentially curative surgery, as majority present at an advanced stage due to lack of effective screening strategies [5]. Despite resection, high recurrence rates of 50-60% persist, conferring a five-year overall survival (OS) of only 30% [5, 6]. The high rate of relapse prompted a strong rationale for adjuvant therapies to improve survival. However, the available evidence remains conflicting as randomized adjuvant trials are still ongoing. A meta-analysis of 6,712 biliary tract cancer (BTC) patients who received varying forms of adjuvant therapy (chemotherapy, radiotherapy, chemoradiotherapy) demonstrated no clear survival benefit with adjuvant treatment (Odds ratio (OR) 0.74, 95% Confidence interval (CI) 0.55-1.01; *P* = 0.06) [7]. Liver transplantation, though not considered as standard therapy for CCA, has also been explored in selected patients with early stage perihilar CCA, where complete resection is impossible due to vascular or biliary invasion. A meta-analysis of 605 CCA patients who underwent liver transplantation demonstrated a 5-year OS of 39%, with superior outcomes in those who underwent perioperative chemoradiotherapy (5-year OS 57%) [8].

Majority of the patients present at an advanced stage, with limited treatment options which include locoregional or systemic therapy. There has been a growing interest in various locoregional therapy modalities including transarterial chemoembolization, selective internal radiotherapy, external beam radiation or ablation in patients who present with liver-limited disease [9]. However, these therapies were evaluated in small retrospective series or single arm phase II trials, and thus limit generalizability. The current standard of care for first line treatment of unresectable CCA is the combination of gemcitabine and cisplatin, albeit with modest benefit [10]. The prognosis of patients with unresectable or metastatic CCA is universally poor, with a median OS of less than one year. The treatment complexity is further confounded by the presence of recurrent cholangitis or cholestasis, necessitating interventions for restoration of biliary drainage and long term antibiotics use, thus leading to delays in systemic treatment.

Notably, the conduct of phase III randomized controlled trials (RCTs) have been exceptionally challenging due to the rarity of CCA and its inherent anatomical, pathological and molecular heterogeneity. With the advent of whole genome sequencing, mutations in epidermal growth factor receptor (*EGFR*), Kirsten rat sarcoma viral oncogene homolog (*KRAS*), v-raf murine sarcoma viral oncogene homolog (*BRAF*) and tumor protein p53 *(TP53*) were unraveled. More recently, novel mutations in isocitrate dehydrogenase (*IDH*), BRCA1-Associated Protein 1 (*BAP1*) and AT-rich interactive domain-containing protein 1A (*ARID1A*), and novel fusions such as fibroblast growth factor receptor (*FGFR2*) and ROS proto-oncogene 1 (*ROS1*) were revealed. In this review, we will discuss the evolving genetic landscape and summarize the targeted therapies in CCA.

## SYSTEMIC CHEMOTHERAPY

The standard of care for first line chemotherapy for advanced CCA is the combination of gemcitabine and cisplatin. The pivotal United Kingdom National Cancer Research Institute Advanced Biliary Cancer (ABC)-02 study reported superior survival with gemcitabine and cisplatin (GC), with a median OS of 11.7 months *versus* 8.1 months, and median progression free survival (PFS) of 8.0 months *versus* 5.0 months, when compared to gemcitabine alone [10]. Despite intensified evaluation of other chemotherapy combinations with fluorouracil, oxaliplatin or irinotecan, the improvement in survival has been marginal [11]. Currently, there is no standard second-line chemotherapy. In a systemic review of 761 patients, treatment with second-line chemotherapy attained a mean OS of 7.2 months (95% CI 6.2-8.2), PFS of 3.2 months (95% CI 2.7-3.7), response rate (RR) of 7.7% (95% CI 6.5-8.9) and disease control rate (DCR) of 49.5% (95% CI 41.4-57.7) [12]. However, these results need to be interpreted with caution. First, patients who receive second-line chemotherapy have better performance status, which may be associated with improved prognosis [13]. Second, only 15-25% of patients will be fit enough to receive second-line treatment [14]. Third, no RCTs have been included in this systemic review. Given the marginal advances with chemotherapy, emphasis has been shifted to molecularly targeted therapies, either as a single agent or in combination with chemotherapy.

## CURRENT GENETIC LANDSCAPE

CCA represents a molecularly diverse subgroup of BTCs. Genomic profiling with whole-exome and next-generation sequencing has identified multiple molecular aberrations that contribute to its multistep carcinogenesis [15–17]. Well established genomic alterations include overexpression of *EGFR* (5%-27%), vascular endothelial growth factor (*VEGF*) and its receptor (*VEGFR*) (55%-60%), human epidermal growth factor receptor 2 (*HER2*)/erb-b2 receptor tyrosine kinase 2 (*ERBB2*) (0%-20%) [15–19], and MET proto-oncogene (*MET*) (7%-21%) [15, 17, 19, 20], mutations in *BRAF* (5%) and loss of function mutation in *TP53* (3%-45%) [15–17, 21]. Dysregulation of a plethora of key signaling pathways such as *RAS*/*RAF*/mitogen-activated extracellular signal regulated kinase (*MEK*)/extracellular signal-regulated kinases (*ERK*) and phosphatidylinositol 3-kinase (*PI3K*)/phosphatase and tensin (*PTEN*)/protein kinase B (*AKT*)/mechanistic target of rapamycin (*MTOR*) further contribute to its malignant transformation [15–17, 21]. The first whole exome sequencing study of 8 liver-fluke related CCA identified 206 somatic mutations in 187 genes, including novel genes (e.g. *SMAD4* (16.7%), roundabout guidance receptor 2 (*ROBO2*) (9.3%), *GNAS* (9.3%), *MLL3* (14.8%), Cyclin-dependent kinase inhibitor 2A (*CDKN2A)* (5.6%), paternally expressed 3 (*PEG3*) (5.6%), ring fingers proteins (*RNF*) (9.3%) [22]. Another study with genomic profiling on 209 CCA revealed that *SMAD4* and *TP53* were more frequent in Opisthorchis viverrini related CCA, and *IDH1/2* mutations were more frequent in non-Opisthorchis viverrini related CCA [23]. Furthermore, chromatin remodeling genes such as *BAP1*, *ARID1A*, Protein polybromo-1 (*PBRM1*), and *MLL3* were found to be highly mutated in CCA [24]. Other novel genetic signatures include *IDH* mutations (16%-36%) [15–17, 21, 24–27], *FGFR* (5%-50%) [15–17, 28–31] and *ROS1* fusions (9%) [15, 32]. The prevalence of these genetic aberrations vary widely across studies, anatomical sites and geographically, primarily attributed to the heterogeneity of BTCs, limited sample size, retrospective nature of majority of the studies, and different techniques used to identify the genomic mutations.

**Figure 1:**
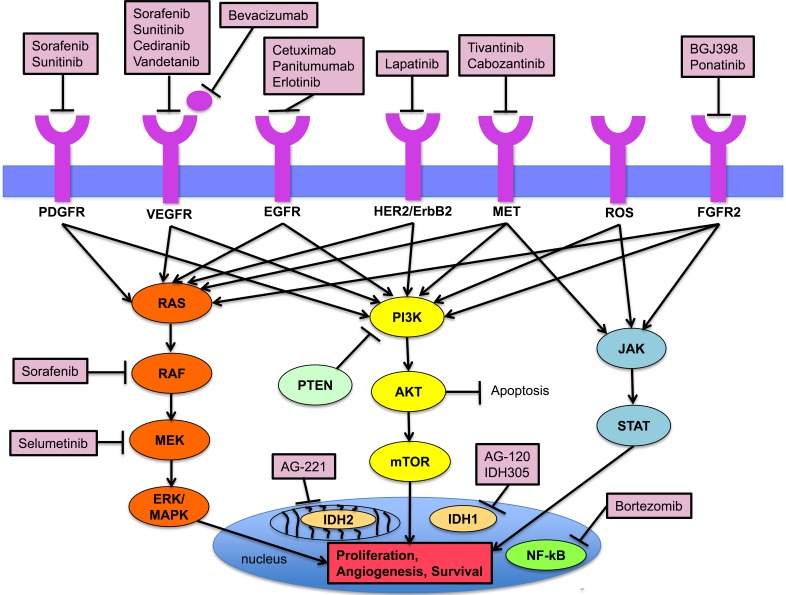
Key signaling pathways in the pathogenesis of cholangiocarcinoma and established targeted agents

Next generation sequencing (NGS) of 46 cancer-related genes in 75 CC patients has highlighted anatomical variability in frequency of mutations [16]. Notably, it may be technically challenging to distinguish ICC and ECC based on pathology, and hence there may be inherent biases in these studies. The common genetic alterations in ICC include *TP53* (30%), *KRAS* (24%), *ARID1A* (20%), *IDH1* (18%) and *MCL1* (16%), whereas for extrahepatic CCA, common aberrations include *TP53* (45%), *KRAS* (40%), *ERBB2* (20%), *SMAD4* (25%), F-box/WD repeat-containing protein 7 (*FBXW7*) (15%) and *CDKN2A* (15%). Furthermore, there were significant differences with regards to the prognostic significance of the above molecular markers, with *TP53*, *KRAS* and *MTOR* alterations predicting a worse prognosis in ICC, and *BAP1*, *PBRM1* and chromatin modulating genes linked to a worse survival in ECC. A subsequent meta-analysis of 4,458 patients with the study of 102 individual markers revealed that genetic alterations of *HER2* and *TP53* were more common in ECC, and *BCL-2*, *EGFR*, *SMAD4*, *p16* and *VEGF-A* were more frequent in ICC [33]. Table 1 summarizes the molecular aberrations in CCA. In the following section, we will highlight the known molecular aberrations in conjunction with their targeted therapies. Figure 1 depicts the key signaling pathways in the pathogenesis of CCA, and novel targeted therapies in development in CCA.

**Table 1:** Molecular aberrations in cholangiocarcinoma

	Intrahepatic cholangiocarcinoma	Extrahepatic cholangiocarcinoma	Reference
*EGFR* overexpression	11%-27%	5%-19%	[15–19]
*KRAS* mutation	9%-24%	40%	[15–17]
*HER2* overexpression	0%-2%	5%-20%	[15–19]
*VEGF* overexpression	54%	59%	[15–19]
*PIK3CA* mutation or deletion	4%	NR	[15–19]
*BRAF* mutation	5%	NR	[21]
*MET* overexpression	7%-21%	0%	[15, 17, 19]
*IDH1/IDH2* mutation	16%-36%	0%	[15–17, 21, 24–27, 54]
*FGFR* translocations	6%-50%	0-5%	[15–17, 28–30, 60]
*TP53* mutation	3%-36%	45%	[15–17]
*ARID1A* mutation	19%-36%	5%	[16, 17, 24]
*MCL1* amplification	16%-21%	NR	[16, 17]
*PTEN* mutation	1%-11%	NR	[15, 17, 21]
*PBRM1* mutation	11%-17%	5%	[16, 24]
*BAP1* mutation	9%-25%	10%	[16, 24]
*SMAD4* mutation	4%	25%	[16]
*FBXW7* mutation	6%	15%	[16]
*CDKN2A* mutation	7%	15%	[17]
*CDK6* mutation	7%	NR	[17]
*BRCA* mutation	4%	NR	[17]
*NF1* mutation	4%	NR	[17]
*TSC1* deletion	4%	NR	[17]
*ROS1* fusion	8.7% (all CCA)	NR	[15, 32]

Abbreviations: NR, not reported

## ESTABLISHED MOLECULAR ABERRATIONS AND TARGETED THERAPY

### EGFR/HER2

The *EGFR* family comprise of *ERBB*1-4, with *ERBB1* (*EGFR*) and *ERBB2* (*HER2*) being frequently implicated in the multi-step carcinogenesis of CCA [15]. Binding of *EGF*-ligands to the receptors induce homodimerization or heterodimerization, which in turn activates downstream signaling pathways (*MAPK, PI3K/AKT/MTOR* and *STAT*) that regulates cell differentiation, migration, angiogenesis and survival. *EGFR* overexpression occurs in 11-27% of ICC and 5-19% of ECC, and has been associated with tumor recurrence and worsened survival [15, 17, 18]. Majority (77-79%) of *EGFR* overexpression in BTCs exhibit copy number gain, with activating mutations in *EGFR* being extremely rare [15]. Although no mutations have been reported in *HER2*, *HER2* overexpression has been noted in 0-2% of ICC and 5-20% of ECC [18]. Preclinical studies have demonstrated that overexpression of *HER2* in transgenic mouse models and orthotopic transplantation BDEneu models enhance the development of CCA, and provided consistent evidence of the oncogenic potential of *EGFR* [34].

Despite the strong rationale of targeting *EGFR* in BTCs and early interesting results with single arm phase II trials suggesting the benefits of *EGFR* inhibitors either as single agents or in combination with chemotherapy (Table 2), four completed randomized studies have failed to confirm the benefits of targeting *EGFR* in advanced BTCs. The only phase III trial of 133 patients with BTCs demonstrated that the addition of erlotinib to gemcitabine-oxaliplatin (GEMOX) significantly improved RR, but did not demonstrate any benefit in survival, with a median OS of 9.5 months in both arms [35]. However, subgroup analyses showed that for patients with CCA, the addition of erlotinib to chemotherapy significantly prolonged median PFS by 2.9 months [5.9 months *vs*. 3.0 months (HR 0.73, 95% CI 0.53-1.00; *P* = 0.049)]. In a phase II study, the addition of cetuximab, a chimeric anti-*EGFR* monoclonal antibody to GEMOX did not confer a survival benefit in patients with advanced BTCs [36]. The median PFS was 6.1 months for the GEMOX and cetuximab arm, compared to 5.5 months in the GEMOX alone arm, and the median OS was 11.0 months and 12.4 months, respectively. In another study, patients who were stratified by *KRAS* status, received GEMOX with or without cetuximab [37]. The addition of Cetuximab to GEMOX was associated with a trend in improvement in PFS (6.7 months *vs*. 4.1 months; *P* = 0.05), but not OS (10.6 months *vs*. 9.8 months; *P* = 0.91). In addition, *KRAS* mutation did not predict for benefit in survival. The addition of another *EGFR* antibody, panitumumab to gemcitabine/cisplatin based chemotherapy did not improve survival in patients with advanced BTCs [38]. Additional biomarker-driven trials will provide further insight as most of the studies were conducted in patients who were unselected for *KRAS* mutation status or other signatures implicated in predicting response to *EGFR* therapy.

**Table 2:** Clinical trials of targeted therapies in biliary tract cancers (including cholangiocarcinoma)

Drug	Study	Phase	Line of Rx	No. of pts	RR (%)	Median PFS (mths)		Median OS (mths)
**Phase III study**								
GEMOX + Erlotinib (A) vs. GEMOX (B)	Lee et al. [35]	III	1st	268	A: 30 B: 16	A: 5.8 B: 4.2		A: 9.5 B: 9.5
**Phase I/II studies**								
*EGFR*								
Erlotinib	Phillips et al. [97]	II	1st/2nd	42	8	2.6	7.5	
Sorafenib + Erlotinib	El-Khoueiry et al. [42]	II	1st	30	7	2	6	
GEMOX + Cetuximab	Gruenberger et al. [98]	II	1st	30	63	8.8	15.2	
GEMOX + Cetuximab	Paule et al. [99]	II	2nd	9	33	Low EGFR: 4 High EGFR: 7	Low EGFR: 7 High EGFR: 9	
GEMOX + Cetuximab (A) vs. GEMOX (B)	Malka et al. [36]	II	1st	150	A: 23 B: 29	A: 6 B: 5.3	A: 11 B: 12.4	
GEMOX + Cetuximab (A) vs. GEMOX (B)	Chen et al. [37]	II	1st	122	A: 27 B: 15	A: 6.7 B: 4.1	A: 10.6 B: 9.8	
Gemcitabine/Capecitabine/Cetuximab	Rubovszky et al. [100]	II	Any	34	17.6	8.6	15.7	
Gemcitabine/Cetuximab	Borbath et al. [101]	II	1st	44	20.4	6 month PFS: 47%	13.5	
GEMOX/Capecitabine/Panitumumab	Jensen et al. [102]	II	Any	46	33	8.3	10	
GEMOX + Panitumumab(KRAS WT)	Hezel et al. [103]	II	1st	31	45	10.6	20.3	
Gemcitabine/irinotecan/Panitumumab	Sohal et al. [104]	II	1st	21	43	NR	12.7	
*HER-2*								
Lapatinib	Ramanathan et al. [105]	II	1st/ 2nd	17	0	1.8	5.2	
*VEGF*/ *VEGFR*								
GEMOX + Bevacizumab	Zhu et al. [39]	II	1st/ 2nd	35	40	7	12.7	
Bevacizumab + Erlotinib	Lubner et al. [40]	II	1st	49	12	4.4	9.9	
Gemcitabine + Capecitabine + Bevacizumab	Iyer et al. [41]	II	1st	50	72	8.1	11.3	
Sorafenib	El-Khoueiry et al. [43]	II	1st	31	0	3	9	
Sorafenib	Bengala [45]	II	Any	46	2	2.3	4.4	
Gemcitabine + Sorafenib (A) vs. Gemcitabine (B)	Moehler et al. [44]	II	1st	102	A: 8 B: 6	A: 3 B: 4.9	A: 8.4 B: 11.2	
Gemcitabine/Cisplatin +Sorafenib	Lee et al. [46]	II	1st	39	NR	6.5	14.4	
Sunitinib	Yi et al. [47]	II	2nd	56	9	1.7	4.8	
Gemcitabine/cisplatin + Cediranib (A) vs. Gemcitabine/cisplatin (B)	Valle et al. [49]	II	1st	124	A: 44 B: 19	A: 8 B: 7.4	A: 14.1 B: 11.9	
Vandetanib	Santoro et al. [48]	II	1st	173	4	105 days	228 days	
*C-MET*								
Tivantinib + Gemcitabine	Pant et al. [52]	I	Any	20	20	NR	NR	
Cabozanitib	Goyal et al. [53]	II	2nd & beyond	19	0	1.8	5.2	
Others								
Selumetinib	Bekaii-Saab et al. [80]	II	1st/ 2nd	28	12	3.7	9.8	
Selumetinib + Gemcitabine/cisplatin	Bridgewater et al.[81]	I	1st	12	37.5% (8 evaluable pts)	6.4	NR	
Bortezomib	Denlinger et al. [85]	II	2nd/ 3rd	20	5	1.6	9.5	

Abbreviations: PFS, Progression free survival; OS, Overall survival; Rx, Treatment ; NR, Not reported; Pts, Patients

### VEGF

The most potent angiogenic factor in perpetuating tumor growth and metastasis is the vascular endothelial growth factor. *VEGF* overexpression was observed in 54% of ICC and 59% of ECC, and has been shown to promote metastasis, tumor recurrence and confer a worse prognosis [15, 18].

The efficacy of *VEGF* inhibitors has been investigated in several trials (Table 2). Bevacizumab has been combined with GEMOX, erlotinib or gemcitabine and capecitabine, yielding a PFS of 4-8 months and OS of 10-13 months [39–41]. Five trials have investigated sorafenib, a multikinase inhibitor against *VEGFR-2*, *VEGFR-3*, *RAF*, platelet derived growth factor receptor (*PDGFR*) and stem cell factor (*KIT*), and did not report any significant benefit in survival [42–46]. Other *VEGF* inhibitors such as sunitinib [47] and vandetanib (ZD6474) [48] yielded disappointing results. Recently, Valle and colleagues reported the results of ABC-03 trial in which the addition of cediranib, a potent oral *VEGFR* 1-3 inhibitor, was evaluated in combination of gemcitabine/cisplatin in advanced BTCs in a randomized phase II trial [49]. Of the 124 patients enrolled (62 in each arm), the addition of cediranib improved the response rate (44% in the cediranib arm and 19% in the placebo arm, *P* = 0.004) but did not improve the median PFS (8.0 months in cediranib arm and 7.4 months in placebo arm, HR 0.93, *P* = 0.72) or OS (14.1 months in cediranib arm and 11.9 months in placebo arm, HR 0.86, *P* = 0.44). Whether other antiangiogenic agents have any benefits in BTCs and whether any biomarkers have any predictive values in BTCs remain to be investigated.

### MET

Binding of hepatocyte growth factor (*HGF*) to *HGF* receptor (*c-MET*) activates multiple key downstream signaling pathways such as the *RAS/MAPK, PI3K/AKT* and *JAK/STAT*, which play critical roles in tumor proliferation and survival [50]. Activation of *MET* can arise *via* mutations or copy number amplification. Through gene expression profiling, increased *c-MET* expression was observed in 20-60% of ICC and 0-70% of ECC [20, 50]. Accumulating evidence has established that *MET* overexpression is associated with a poor prognosis. There is emerging evidence that suggest *MET* aberration to be one of the mechanisms responsible for *EGFR* resistance [51]. This led to the evolution of *MET* inhibitors for CCA, either alone or in combination with cytotoxic agents.

The combination of Tivantinib (ARQ 197) with gemcitabine was examined in 74 patients with solid tumors, with 20% (1 CCA patient) achieving partial response [52]. In another study, 19 CCA patients who were unselected for *MET* amplification or overexpression were treated with cabozantinib and exhibited no objective responses [53]. PFS and OS were 1.77 (95% CI 1.63-5.37) and 5.2 (95% CI 2.70-8.17) months, respectively.

## NOVEL ONCOGENIC DRIVERS

The advent of next generation sequencing techniques has further shaped the genomic landscape of CCA and enhanced our understanding of its pathogenesis. Recent discoveries include *IDH1/2* mutations, *FGFR2* and *ROS1* fusions, and mutations in chromatin remodeling genes for example *ARID1A* and *BAP1*. We will further elaborate on these promising molecular targets.

### IDH mutations

*IDH1* and *2* alterations exist in several tumors including gliomas and more recently identified in BTCs through high throughput molecular profiling [15–17, 21, 25–27, 54]. *IDH1* and *IDH2* are metabolic enzymes that catalyze the oxidative decarboxylation of isocitrate to alpha-ketoglutarate [55]. *IDH* mutations enhance the conversion of alpha-ketoglutarate to 2-hydroxyglutarate (2-HG), an oncometabolite that inhibits α-ketoglutarate-dependent enzymes responsible for DNA methylation, epigenetic regulation and call signaling. The accumulation of 2-HG in tumor tissue in turn promotes cell proliferation and survival.

The frequency of *IDH* mutations ranges from 16-36%, and is ubiquitously higher in ICC than ECC [15–17, 25–27, 54, 55]. *IDH* mutations were observed in 22-36% of ICC and only 0-7% of ECC, and may be associated with clear cell or poorly differentiated histology [26, 55]. The prognostic significance of *IDH* mutations remains conflicting. In a cohort of 326 patients with resected ICC, *IDH* mutation was associated with longer time to recurrence and OS [27]. In addition, the authors observed enhanced *p53* and DNA hypermethylation among patients with *IDH* mutations. In contrast, Jiao et al. demonstrated in a study of 32 patients with ICC that *IDH* mutations confer a worse prognosis when compared to those with *IDH* wild-type (3-year OS 33% *vs*. 81%; *P* = 0.003) [24]. However, this adverse finding may be due to the presence of a larger proportion of stage IV disease amongst the *IDH* mutants compared to *IDH* wild-type (50% *vs*. 15%). Two recent studies revealed no correlation between *IDH* mutation status and survival among 200 patients with resected ICC [21] and 104 patients with advanced ICC [54].

Two proof of concept studies illustrated the tumor suppressive effects of *IDH* inhibitors. Rohle et al. found that a selective R132H-IDH1 inhibitor (AGI-5198) impeded the growth of *IDH*-mutant glioma cells [56]. Similarly, Wang et al. showed that AGI-6780 selectively inhibits the leukemic cells harboring mutant *IDH2*/R140Q [57]. Current *IDH*-inhibitor studies are in early clinical development (NCT02073994, NCT02381886 and NCT02273739). The preliminary results of a phase 1 trial of AG120 (*IDH1* inhibitor) in 62 patients with *IDH1* mutation positive solid tumors who had progressed on standard treatment was reported at the AACR-NCI-EORTC International Conference on Molecular Targets and Cancer Therapeutics 2015. There were no dose limiting toxicities, with anemia being the most frequent Grade 3 AE (5%). 1/20 (5%) CC patients attained PR and 11/20 (55%) attained SD. Reduction in circulating 2-HG level was observed ranging from 73% to 99%, and reduction in Ki67 staining was seen from 22% - 96%. The expansion phase with 500 mg QD is underway (NCT02073994).

### Fibroblast growth factor receptor (FGFR) 2 fusions

*FGFR2*, a member of the fibroblast growth factor family of receptors (*FGFR 1-4*), is located at chromosome 10q26 and mitigates cell differentiation, proliferation and apoptosis [58]. The oncogenic property of *FGFR2* has been linked to loss of the carboxy terminus and ligand independent dimerization, leading to *FGFR* protein overexpression.

Whole exome sequencing and fluorescence *in situ* hybridization (FISH) have identified *FGFR2* alterations primarily in 6%-50% of ICC and 0-5% of ECC [28–31, 59, 60]. Churi et al. analyzed 75 CCA patients with next generation sequencing, and found that genetic alterations in the *FGFR* pathway occurred in 13% of intrahepatic CCA and 5% of extrahepatic CCA, and that these alterations were associated with improved survival [16].

More recently, *FGFR2* fusions have been detected in several studies (Table 3). These fusions are a product of the *FGFR* receptor (exons 1-19) and various partners (e.g. *AHCYL1*, *BICC1, KCTD1* and *TXLNA)*. The fusion protein is activated by the enforced dimerization of the respective partners with resultant intracellular domain tyrosine residue phosphorylation, and activation of downstream signaling pathways including *MAPK*, *PIK3/AKT/MTOR* and *JAK/STAT* pathways [59]. There are marked variability in the frequency of *FGFR2* fusions, ranging from 6-50% in ICC, and rarely in ECC. In a series of 102 patients with BTCs, Arai et al. observed *FGFR2* fusions (*FGFR2-AHCYL1* or *FGFR2-BICC1*) in 13.6% of ICC (9/66 ICC), and that inhibition of *FGFR2* impeded activation of *MAPK* pathway, which is responsible for uncontrolled tumor growth [28]. Another study evaluated 152 CCA and 4 intraductal papillary biliary neoplasm of the bile duct with FISH, and reported *FGFR2* translocation in 12/96 (13%) of ICC, with a female predominance [29]. Those who harbored *FGFR2* translocations had improved cancer-specific survival (123 *vs*. 37 months) and superior DFS (125 months *vs*. 26 months). Furthermore, cholangiocarcinoma harboring *FGFR2* translocation and concomitant *KRAS* mutation are only rarely reported [31]. Therefore, this association remains to be explored in larger cohorts to further assess if *FGFR2* translocation work in synergy with *KRAS* mutation in promoting carcinogenesis in CCA. In a study comprising of 109 ICC, 40 ECC and 11 gallbladder cases, novel *FGFR2* gene fusions (*FGFR2-KCTD1* and *FGFR2-TXLNA)* and a new variation of *FGFR2-BICC* (Type 2) were reported [60]. Using NIH3T3 clones that express either wild-type or kinase-inactive mutant forms of *FGFR2-KCTD1* or *FGFR2-TXLNA*, the Nakamura et al. showed that wild-type *FGFR* fusions, and not the mutant forms induce tumor growth *in vivo via* ligand-independent autophosphorylation and activation of the *MAPK* signaling pathway. In addition, there was marked inhibition of *FGFR* autophosphorylation and cell proliferation by the *FGFR* inhibitors (BGJ398 and PD173474).

**Table 3:** *FGFR2* translocations in ICC

Study	No. of patients (n)	No. of patients with FGFR2 translocation (n, %)	Type of FGFR2 Translocations	Method
Wu et al. [59]	2	2 (100%)	*FGFR2-BICC1*	RNA, exome sequencing
Borad et al. [30]	6	3 (50%)	*FGFR2-TACC3* *FGFR2-BICC1* *FGFR2-MGEA5*	Genome-wide and whole transcriptome sequencing
Graham et al. [29]	96	12 (13%)	NR	Fluorescence in situ hybridization
Arai et al. [28]	66	9 (13.6%)	*FGFR2-AHCYL1* *FGFR2-BICC1*	Whole transcriptome sequencing
Ross et al.[17]	28	3 (10.7%)	*FGFR2-KIAA1598* *FGFR2-BICC1* *FGFR2-TACC3*	Next generation sequencing
Sia et al. [31]	107	48 (45%)	*FGFR2-PPHLN1* *FGFR2-BICC1*	RNA, exome sequencing
Nakamura et al. [60]	109	6 (5.5%)	*FGFR2-KCTD1* *FGFR2-TXLNA* *FGFR2-BICC1(Type 2)*	Exome sequencing

Abbreviations: NR, Not reported

In a genome-wide and whole transcriptome sequencing on 6 ICC samples with *FGFR2* translocations in 3/6 (50%) patients, two out of three patients responded to *FGFR2* inhibitors [30]. One patient with *FGFR2-MGEA5* fusion was treated with ponatinib (a pan-*FGFR* inhibitor) and had a biochemical CA 19-9 response with shrinkage of tumor. Another patient with *FGFR2-TACC3* fusion who previously achieved a partial response with pazopanib, and subsequently received ponatinib attained stable disease. These encouraging results suggest that *FGFR2* has the potential to be an actionable molecular target, and that patients who harbor these alterations may benefit from tyrosine-kinase directed therapies. An ongoing phase 2 study of BGJ398 (a selective pan-*FGFR* inhibitor) in patients with advanced or metastatic CCA with *FGFR* genetic alterations reported promising efficacy (Javle MM et al, 2016 Gastrointestinal Cancer Symposium, J Clin Oncol 34, 2016 (suppl 4S; abstr 335)) The overall RR was 22% (8/36 evaluable patients) and DCR was 75% (27/26 patients). BGJ398 was generally well tolerated. The Grade 3/4 AEs include hyperphosphatemia (19%), hypophosphatemia (9%), hyponatremia (6%), and asymptomatic increased lipase (6%). This is a promising drug that warrants further investigation.

## IMMUNE CHECKPOINT INHIBITORS

Immune checkpoints including cytotoxic T-lymphocyte-associated antigen (CTLA)-4, programmed cell death (*PD*)-1 receptor and its ligands (*PD-L1*, *PD-L2*) promotes T-cell anergy [61]. Increased levels of tumor-infiltrating CD8+ cytotoxic T cells and/or CD4+ T cells have been shown to be associated with improved prognosis in BTCs [62]. Given the success of ipilimumab (CTLA-4 monoclonal antibody), pembrolizumab and nivolumab (anti-*PD-1* antibodies) in the treatment of metastatic melanoma [63, 64], there has been growing interest of the benefit of immunomodulation in BTCs. In a preclinical study of intrahepatic CCA, Koido et al. showed that both gemcitabine and interferon −γ led to an upregulation of *PD-L1*, which suggest that treatment with *PD-L1* blockade may be beneficial [65]. Studies have suggested that mismatch repair (MMR) deficient tumors are more responsive to PD-1 blockade than are MMR proficient tumors [66]. A phase II study demonstrated that pembrolizumab led to high RR in colorectal cancer patients with genetic defects in mismatch repair (MMR) [66]. The phase II study with pembrolizumab in MMR deficient non-colorectal gastrointestinal cancers (ampullary (*n* = 4), pancreas (*n* = 4), biliary (*n* = 3), small bowel (*n* = 3), and gastric (*n* = 3) cancers) is ongoing. An interim analysis reported an ORR of 50% and DCR of 70% in 10 evaluable patients. The OS was 21 months and PFS was not reached (Le DT et al, 2016 Gastrointestinal Symposium, J Clin Oncol 34, 2016 (suppl 4S; abstr 195)). There are currently no studies evaluating the efficacy of *PD-1* inhibitors in CCA patients with microsatellite instability (MSI)-high *versus* MSI-stable tumors. The interim results of another phase 1b study of pembrolizumab (MK-3495) in patients with advanced BTC was presented at the European Cancer Congress 2015 (NCT02054806). Pembrolizumab was well tolerated with an ORR of 17.4% (95% CI, 5.0-38.8) in the 23 evaluable patients. 4/24 (16.7%) of the patients experienced a treatment-related grade 3 AE (anemia, autoimmune hemolytic anemia, colitis, and dermatitis). Currently, pembrolizumab is evaluated in combination with mFOLFOX6 in a phase 1/2 study at the University of Utah (NCT02268825).

## LESS-ESTABLISHED MOLECULAR ABERRATIONS

There has been limited studies regarding the following molecular aberrations and additional studies are required to provide further insight.

### ROS1

Elevated *ROS* expression has been observed in non-small cell lung cancer, glioblastoma and breast cancer [32]. *ROS* kinase fusions [between kinase domain of *ROS* and Fused in Glioblastoma (*FIG*) gene] has been described in 8.7% of patients with CCA [32]. These fusions further activate downstream effectors such as *STAT3* and *AKT*. The *FIG-ROS* fusion driver gene has been shown to accelerate tumor growth in an orthotopic allograft mouse model, and that inactivation of the gene portends an antitumor effect [67].

Notably, TAE684 (an *ALK* inhibitor) has been shown to inhibit *ROS* kinase activity, with consequent cell inhibition and cell death in BaF3 cells expressing this fusion protein [32]. Given the success of crizotinib in attaining an impressive response rate of 48% in *ROS1*- rearranged non-small cell lung cancer [68], similar studies in CCA are warranted to evaluate the potential benefit of targeted therapy in patients with *ROS* fusions. A phase II trial of crizotinib in patients with *ALK*, *MET* or *ROS1* alterations is underway (NCT02034981).

### PI3K/AKT/MTOR

Constitutive activation of the *EGFR*, *HER2*, *MET* and Insulin growth factor (*IGF*) receptor or disruption of the *PTEN* and *SMAD4* triggers the downstream activation of *PI3K/PTEN/AKT/mTOR* signaling pathway [69, 70]. Dysregulation of this pathway subsequently stimulates cell proliferation, angiogenesis and survival. Activation of this pathway in patients harboring *EGFR*, *HER2* and *MET* overexpression has been reported in as high as 65% of tumors. The incidence of *PIK3CA* (a subunit of PI3K) hotspot mutations in CCA ranges from 5% to 34% [71]. Furthermore, increased expression of phosphor-*AKT1* and phosphor-*MTOR* in intrahepatic CCA is positively correlated with prognosis and that this association was not modified by *PTEN* expression [72].

Dual inhibition of *AKT* and *MTOR* with MK-2206 and everolimus (RAD001) has been shown to enhance anti-proliferative effects in CCA [73]. More recently, increased efficacy was attained in-vitro by dual inhibition of the *PI3K/AKT/MTOR* and *RAF/MEK/ERK* pathway, which overcame resistance pathways [74]. A phase I trial of mFOLFOX6 and the oral *PI3K* inhibitor BKM120 in patients with advanced solid tumors (4/17 CCA) reported high toxicity rates, with 76 % of the patients experiencing a grade 3/4 AE [75]. The most common AEs were neutropenia, fatigue, leukopenia, hyperglycemia and thrombocytopenia. 1/4 of the CCA patients achieved SD. The combination of everolimus with gemcitabine and cisplatin was evaluated in 10 CCA and gallbladder cancers, of which 60% had SD [76]. Currently, MK2206 (*AKT* inhibitor) is being investigated in advanced refractory BTC (NCT01425879).

### RAS/RAF/MEK/ERK

The *RAS/RAF/MEK/ERK* signal transduction pathway is frequently dysregulated in BTCs [77]. Activation of this pathway requires the binding of *EGF*, *PDGF* and cytokines to its receptors, with subsequent transactivation of downstream signaling cascade, leading to the end-phosphorylation of *MEK1* and *2* and *ERK-1* and *ERK-2*. *MEK* is an attractive target as *ERK -1* and *ERK-2* are the only known *MEK* substrates [70]. Gain of function mutations in *KRAS* constitutes one of the most frequent mutations in CCA, with the most frequent alteration in codon 12 [15]. The frequency of activating *KRAS* mutations ranges from 9%-40% [15–17]. *KRAS* has been associated with perineural invasion and poor prognosis [78]. In addition, there is marked anatomical variability in *KRAS* mutation, with *KRAS* mutations observed in 53.3% of perihilar-type, but only 16.7% of intrahepatic CCA. Notably, the incidence of *KRAS* mutations increases with disease stage.[79] Despite the recognized frequency of *KRAS* mutations, targeting this pathway remains challenging. Early evidence of efficacy of *MEK* inhibitor was reported in a single arm study of selumetinib in advanced BTCs [80]. Of the 28 patients enrolled, 3 patients had confirmed partial responses. In this study, no *BRAF* V600E mutations were found. Recently, the ABC-04 study of selumetinib in combination with gemcitabine and cisplatin in advanced or metastatic BTC (9/13 CCA) demonstrated a RR of 37.5%, a median PFS of 6.4 months and manageable toxicities [81].

### BRAF

*B-Raf* is a proto-oncogene and is a key component of the *RAS/RAF/MEK/ERK* proliferation signaling pathway. The most common *BRAF* gene mutation found in human cancers is V600E, and exists in up to 22% of CCA in one report [82]. More importantly, *BRAF* and *KRAS* mutations are mutually exclusive. In a recent phase II “basket” study of vemurafenib in *BRAF* V600 mutated non-melanoma cancers, one patient with CCA achieved a durable PR of more than one year [83].

### NFk-B

Several studies have suggested the *NF-kB*, a transcriptional nuclear factor, plays a critical role in tumor migration and treatment resistance in several tumors, although the evidence is not conclusive [84]. This stems from the observation that tumor proliferation can be kept in check *via* proteasome inhibition, which halts the clearance of pro-apoptotic factors. To date, the only proteasome inhibitor investigated was bortezomib and results were disappointing, with no objective response, median time to progression was 5.8 months and median OS was 9 months [85].

### JAK/STAT cytokine pathway

Binding of pro-inflammatory cytokine, interleukin-6 (IL-6) to gp130 triggers the downstream activation of the *JAK/STAT* pathway, leading to the silencing of its inhibitor, suppressor of cytokine signaling-3 (*SOCS3*) [86]. This in turn accelerates inflammation, cell growth and tumor formation. This pathway has been noted in 70% of the inflammation subclass in ICC, characterized by activation of the *STAT3* and cytokine pathways and improved prognosis.[87] Furthermore, the *JAK2* inhibitor AZD1480 has been demonstrated to inhibit Stat3 signaling and exhibit anti-tumor efficacy in solid tumor cell lines [88].

### Notch signaling pathways

The *Notch* signaling cascade is a highly conserved pathway, responsible for cell differentiation, apoptosis and cell survival. To date, there are four known *Notch* receptors and five ligands. Aberrant *Notch* signaling was first described in acute T-cell lymphoblastic leukemia, and subsequently in CCA [89, 90]. *Notch*-mediated conversion of hepatocytes into biliary lineage has been shown to promote intrahepatic CCA formation and progression in a mouse model of ICC [91]. *Notch* 1 and 4 were noted to be more frequently expressed in tumor cells compared to normal tissue. The frequency of *Notch* expression in ICC for *Notch* 1, *Notch* 2, *Notch* 3 and *Notch* 4 were 82.9%, 56.1%, 39.0% and 34.1% respectively [92]. In addition, *Notch* 4 was found to be prognostic and *Notch* 1 overexpressed in large tumors. Furthermore, *Notch* overexpression has been demonstrated to predict sensitivity to 5-fluorouracil *in vivo*. The complex *Notch* signaling pathway warrants further understanding before the advent of novel *Notch* targeting agents.

### Protein kinase A regulatory subunit 1 alpha (PRKAR1A) pathway

Protein kinase A is a cyclic AMP (*cAMP)*-dependent protein kinase and is part of the serine-threnonine protein kinase family. Activation of the *PRKAR1A/PKAI* pathway is found in various tumors, including CCA [93]. More recently, fusion genes comprising of *cAMP*-dependent protein kinase (*PKA*) and mitochondrial ATP synthase (*ATP1B-PRKACA* and *ATP1B-PRKACB*) were detected with resultant increased expression of *PRKACA* and *PRKACB* and activation of *MAPK* signaling [60]. The abrogation of *PRKAR1A* gene expression has been linked to significant cell inhibition and apoptosis of CC cells *via* suppression of the *JAK/STAT*, *MAPK*, *PI3K/AKT* and *WNT/β-catenin* pathway signaling. Drug evaluation with *PKA* inhibitor (isoquinoline H89) as well as site-specific *cAMP* analogs (8-Cl *cAMP* and 8-Br *cAMP*) showed promising anti-proliferative effect in CCA cells, supporting the notion that *PKA* can potentially contribute as a drug target in CCA.

### Wnt/β-catenin pathway

Aberrant genetic alterations of the Wingless-type MMTV integration site family (*Wnt*)/*β-catenin* signaling cascade has been implicated in tumorigenesis in several studies [94]. The *Wnt* signaling pathway is highly activated in CCA, and an inflammatory milieu comprising of inflammatory macrophages is required for its sustainability [95]. Furthermore, tumor regression in mouse and rat models were prompted with the introduction of *Wnt* inhibitors. The *Wnt* signaling pathway has also been postulated as one of the mechanisms responsible for chemoresistance in CCA [94]. GSK3β, a “destruction complex” phosphorylates and degrade β-catenin, leading to downregulation of the *Wnt* survival pathway. Recently, Huang et al. showed that β-escin, an active compound in horse chestnut (*Aesculus hippocastanum*) seed, could inhibit the GSK3β/β-catenin pathway and thus terminate cell growth [96]. Hence, the *Wnt* signaling pathway may represent another alternative target for ICC treatment.

Clinical studies with novel agents in early development are summarized in Table 4.

**Table 4:** Targeted therapies in development

Drug	Target	Phase	Line of therapy	NCT number
AG-120	*IDH1*	I	2nd & beyond	NCT02073994
IDH305	*IDH1*	I	2nd & beyond	NCT02381886
AG-221	*IDH2*	I/II	2nd & beyond	NCT02273739
Dasatinib	*IDH1/2*	II	2nd & beyond	NCT02428855
BAY1187982	*FGFR2*	I	2nd & beyond	NCT02368951
ARQ087	*FGFR2*	I/II	2nd & beyond	NCT01752920
BAY1179470	*FGFR2*	I	Any	NCT01881217
AZD4547	*FGFR2*	I	Any	NCT00979134
BGJ398	*FGFR2*	II	2nd & beyond	NCT02150967
Ponatinib Hydrochloride	*FGFR2*	II	Any	NCT02265341
BLU-554	*FGFR4*	I	Any	NCT02508467
Erlotinib + Cetuximab	*EGFR*	I	Any	NCT00397384
GEMOX ± Cetuximab	*EGFR*	II	1st	NCT01267344
GEMOX ± Panitumumab	*EGFR*	II	1st	NCT01389414
GEMOX/Capecitabine ± Panitumumab	*EGFR*	II	Any	NCT00779454
GEMOX ± Panitumumab	*EGFR*	II	1st	NCT01389414
Gemcitabine/cisplatin + BIBW 2992	*EGFR/HER2*	I	1st	NCT01679405
Afatinib + Capecitabine	*EGFR/HER2*	I	2nd & beyond	NCT02451553
ASLAN001	*EGFR, HER2, HER4*	II	2nd & beyond	NCT02609958
Cediranib + mFOLFOX6	*VEGF*	II	1st	NCT01229111
Gemcitabine + Oxaliplatin + Capecitabine + Panitumumab/Bevacizumab	*EGFR, VEGF*	II	1st	NCT01206049
Ramucirumab	*VEGFR*	II	2nd & beyond	NCT02520141
Lenvatinib	*VEGFR*	II	2nd & beyond	NCT02579616
LY2801653	*c-MET*	I	2nd & beyond	NCT01285037
Everolimus	*MTOR*	I	2nd & beyond	NCT00949949
Trametinib	*MEK*	II	2nd & beyond	NCT02042443
MK2206	*AKT*	II	2nd	NCT01425879
LDK378	*ROS1*	II	1st or 2nd	NCT02374489
Ceritinib	*ALK*	II	2nd & beyond	NCT02638909
Sorafenib + GEMOX	*VEGFR,* *PDGFR,* *RAF, KIT*	I/II	Phase 1: Any Phase II: 1st	NCT00955721
Regorafenib	*EGFR, Ras, Raf, VEGFR, PDGFR*	II	2nd	NCT02053376
Regorafenib	*EGFR, Ras, Raf, VEGFR, PDGFR*	II	2nd & beyond	NCT02115542
Pazopanib + GSK1120212	*VEGFR/* *PDGFR* */Raf /MEK*	I	Any	NCT01438554
Gemcitabine + Pazopanib	*c-KIT, FGFR, PDGFR and VEGFR*	II	1st	NCT01855724
Pembrolizumab	*PD-1*	II	2nd & beyond	NCT02628067
Pembrolizumab + mFOLFOX	*PD-1*	I/II	Any	NCT02268825
	*PD-L1*	I	2nd & beyond	NCT01938612
Gemcitabine/Cisplatin ± CX-4945	*CK2*	I/II	1st	NCT02128282
BBI503	Cancer stemness kinase	II	2nd & beyond	NCT02232633
DKN-01 and Gemcitabine/Cisplatin	Dkk-1	I	1st	NCT02375880
ADH-1	*ICAM-1*	I	1st	NCT01825603

Abbreviations: *CK2*, Caesin kinase 2; *ICAM-1*, Intercellular adhesion molecule-1, *Dkk-1*, dickkopf *Wnt* signaling pathway inhibitor 1; mFOLFOX; Modified fluorouracil, folinic acid and oxaliplatin.

## CONCLUSIONS

Advanced CCA portends a dismal prognosis despite standard treatment with gemcitabine and cisplatin. Given the modest benefits with chemotherapy alone and the anatomical, pathological and molecular heterogeneity, there is an unmet and imperative need for comprehensive genomic profiling to improve the understanding of the pathogenesis of CCA, with the aim of personalized treatment. To achieve this aim, we must overcome the mounting challenges, which include a lack of RCTs due to the rarity of CCA, and the inherent complexity due to interactions of the signaling pathways. Extensive collaborative efforts will be required to formulate adequately powered biomarker-driven trials to improve clinical outcomes. Results of the *EGFR* inhibitors have been disappointing. As the majority of the trials are performed in unselected population, it will be informative to conduct trials in patients enriched for the presence of molecular signatures implicated in predicting *EGFR* sensitivity to determine its efficacy. Given the promising early evidence of efficacy signal with *IDH* and *FGFR2* inhibitors in early phase trials, additional studies should focus on novel strategies targeting *IDH* mutations and *FGFR2* fusions. Furthermore, the identification of oncogenic addiction loops, or novel combination strategy that targets critical molecular pathways simultaneously will be paramount to improve the clinical outcome in CCA.

## ABBREVIATIONS

CCA: Cholangiocarcinoma
EGFR: Epidermal growth factor receptor
KRAS: Kirsten rat sarcoma viral oncogene homolog
BRAF: V-raf murine sarcoma viral oncogene homolog
IDH: Isocitrate dehydrogenase
BAP1: BRCA1-Associated Protein 1
ARID1A: AT-rich interactive domain-containing protein 1A
FGFR: Fibroblast growth factor receptor
ROS1: ROS proto-oncogene 1
OS: Overall survival
BTC: Biliary tract cancer
OR: Odds ratio
CI: Confidence interval
RCT: Randomized controlled trials
GC: Gemcitabine and cisplatin
PFS: Progression free survival
HR: Hazard ratio
DCR: Disease control rate
VEGF: Vascular endothelial growth factor
VEGFR: Vascular endothelial growth factor receptor
HER2: Human epidermal growth factor receptor 2
ERBB2: erb-b2 receptor tyrosine kinase 2
MET: MET proto-oncogene
TP 53: Tumor protein p53
MEK: Mitogen-activated extracellular signal regulated kinase
ERK: Extracellular signal-regulated kinases
PI3K: Phosphatidylinositol 3-kinase
PTEN: Phosphatase and tensin
AKT: Protein kinase B
MTOR: Mechanistic target of rapamycin
NGS: Next generation sequencing
PBRM1: Protein polybromo-1
GEMOX: Gemcitabine-oxaliplatin
PDGFR: Platelet derived growth factor receptor
KIT: Stem cell factor
HGF: Hepatocyte growth factor
IGF: Insulin growth factor
SMAD4: Mothers against Decapentaplegic Homolog
IL-6: Interleukin-6
SOCS3: Suppressor of cytokine signaling-3
ROBO2: Roundabout guidance receptor 2
CDKN2A: Cyclin-dependent kinase inhibitor 2A
PEG3: Paternally expressed 3
RNF: Ring fingers proteins
2-HG: 2-hydroxyglutarate
FISH: Fluorescence *in situ* hybridization
PRKAR1A: Protein kinase A regulatory subunit 1 alpha
Wnt: Wingless-type MMTV integration site family
CTLA: Cytotoxic T-lymphocyte-associated antigen
PD: Programmed cell death
PD-L: Programmed cell death ligand
MCL1: Induced myeloid leukemia cell differentiation protein Mcl-1
FBXW7: F-box/WD repeat-containing protein 7
CDK6: Cell division protein kinase 6
NF1: Neurofibromin 1
TSC1: Tuberous sclerosis 1
BRCA: Breast Cancer susceptibility gene
RR: Response rate
CK2: Caesin kinase 2
ICAM-1: Intercellular adhesion molecule-1

## References

[1] Khan SA, Toledano MB, Taylor-Robinson SD (2008). Epidemiology, risk factors, and pathogenesis of cholangiocarcinoma. HPB (Oxford).

[2] Siegel R, Naishadham D, Jemal A (2013). Cancer statistics, 2013. CA Cancer J Clin.

[3] Yang JD, Kim B, Sanderson SO, Sauver JS, Yawn BP, Larson JJ, Therneau TM, Roberts LR, Gores GJ, Kim WR (2012). Biliary tract cancers in Olmsted County, Minnesota, 1976-2008. Am J Gastroenterol.

[4] Zhou Y, Zhao Y, Li B, Huang J, Wu L, Xu D, Yang J, He J (2012). Hepatitis viruses infection and risk of intrahepatic cholangiocarcinoma: evidence from a meta-analysis. BMC Cancer.

[5] Nathan H, Pawlik TM, Wolfgang CL, Choti MA, Cameron JL, Schulick RD (2007). Trends in survival after surgery for cholangiocarcinoma: a 30-year population-based SEER database analysis. J Gastrointest Surg.

[6] Mavros MN, Economopoulos KP, Alexiou VG, Pawlik TM (2014). Treatment and Prognosis for Patients With Intrahepatic Cholangiocarcinoma: Systematic Review and Meta-analysis. JAMA Surg.

[7] Horgan AM, Amir E, Walter T, Knox JJ (2012). Adjuvant therapy in the treatment of biliary tract cancer: a systematic review and meta-analysis. J Clin Oncol.

[8] Gu J, Bai J, Shi X, Zhou J, Qiu Y, Wu Y, Jiang C, Sun X, Xu F, Zhang Y, Ding Y (2012). Efficacy and safety of liver transplantation in patients with cholangiocarcinoma: a systematic review and meta-analysis. Int J Cancer.

[9] Amini N, Ejaz A, Spolverato G, Kim Y, Herman JM, Pawlik TM (2014). Temporal trends in liver-directed therapy of patients with intrahepatic cholangiocarcinoma in the United States: a population-based analysis. J Surg Oncol.

[10] Valle J, Wasan H, Palmer DH, Cunningham D, Anthoney A, Maraveyas A, Madhusudan S, Iveson T, Hughes S, Pereira SP, Roughton M, Bridgewater J, Investigators ABCT (2010). Cisplatin plus gemcitabine versus gemcitabine for biliary tract cancer. N Engl J Med.

[11] Ramirez-Merino N, Aix SP, Cortes-Funes H (2013). Chemotherapy for cholangiocarcinoma: An update. World J Gastrointest Oncol.

[12] Lamarca A, Hubner RA, David Ryder W, Valle JW (2014). Second-line chemotherapy in advanced biliary cancer: a systematic review. Ann Oncol.

[13] Sang-Cheol Lee KK, Hanjo Kim, Kim Hyun Jung, Kim Se Hyung, Sang-Byung Bae, Kim Chan Kyu, Namsu Lee, Lee Kyu Taek, Park Sung Kyu, Jong-Ho Won, Jina Yun, Hong Dae Sik, Park Hee Sook (2012). Prognostic factor analysis of second-line chemotherapy in advanced biliary tract cancer. J Clin Oncol.

[14] Bridgewater J, Palmer D, Cunningham D, Iveson T, Gillmore R, Waters J, Harrison M, Wasan H, Corrie P, Valle J (2013). Outcome of second-line chemotherapy for biliary tract cancer. Eur J Cancer.

[15] Andersen JB (2015). Molecular pathogenesis of intrahepatic cholangiocarcinoma. J Hepatobiliary Pancreat Sci.

[16] Churi CR, Shroff R, Wang Y, Rashid A, Kang HC, Weatherly J, Zuo M, Zinner R, Hong D, Meric-Bernstam F, Janku F, Crane CH, Mishra L, Vauthey JN, Wolff RA, Mills G (2014). Mutation profiling in cholangiocarcinoma: prognostic and therapeutic implications. PLoS One.

[17] Ross JS, Wang K, Gay L, Al-Rohil R, Rand JV, Jones DM, Lee HJ, Sheehan CE, Otto GA, Palmer G, Yelensky R, Lipson D, Morosini D, Hawryluk M, Catenacci DV, Miller VA (2014). New routes to targeted therapy of intrahepatic cholangiocarcinomas revealed by next-generation sequencing. Oncologist.

[18] Yoshikawa D, Ojima H, Iwasaki M, Hiraoka N, Kosuge T, Kasai S, Hirohashi S, Shibata T (2008). Clinicopathological and prognostic significance of EGFR, VEGF, and HER2 expression in cholangiocarcinoma. Br J Cancer.

[19] Nakazawa K, Dobashi Y, Suzuki S, Fujii H, Takeda Y, Ooi A (2005). Amplification and overexpression of c-erbB-2, epidermal growth factor receptor, and c-met in biliary tract cancers. J Pathol.

[20] Miyamoto M, Ojima H, Iwasaki M, Shimizu H, Kokubu A, Hiraoka N, Kosuge T, Yoshikawa D, Kono T, Furukawa H, Shibata T (2011). Prognostic significance of overexpression of c-Met oncoprotein in cholangiocarcinoma. Br J Cancer.

[21] Zhu AX, Borger DR, Kim Y, Cosgrove D, Ejaz A, Alexandrescu S, Groeschl RT, Deshpande V, Lindberg JM, Ferrone C, Sempoux C, Yau T, Poon R, Popescu I, Bauer TW, Gamblin TC (2014). Genomic profiling of intrahepatic cholangiocarcinoma: refining prognosis and identifying therapeutic targets. Ann Surg Oncol.

[22] Ong CK, Subimerb C, Pairojkul C, Wongkham S, Cutcutache I, Yu W, McPherson JR, Allen GE, Ng CC, Wong BH, Myint SS, Rajasegaran V, Heng HL, Gan A, Zang ZJ, Wu Y (2012). Exome sequencing of liver fluke-associated cholangiocarcinoma. Nat Genet.

[23] Chan-On W, Nairismagi ML, Ong CK, Lim WK, Dima S, Pairojkul C, Lim KH, McPherson JR, Cutcutache I, Heng HL, Ooi L, Chung A, Chow P, Cheow PC, Lee SY, Choo SP (2013). Exome sequencing identifies distinct mutational patterns in liver fluke-related and non-infection-related bile duct cancers. Nat Genet.

[24] Jiao Y, Pawlik TM, Anders RA, Selaru FM, Streppel MM, Lucas DJ, Niknafs N, Guthrie VB, Maitra A, Argani P, Offerhaus GJ, Roa JC, Roberts LR, Gores GJ, Popescu I, Alexandrescu ST (2013). Exome sequencing identifies frequent inactivating mutations in BAP1, ARID1A and PBRM1 in intrahepatic cholangiocarcinomas. Nat Genet.

[25] Borger DR, Tanabe KK, Fan KC, Lopez HU, Fantin VR, Straley KS, Schenkein DP, Hezel AF, Ancukiewicz M, Liebman HM, Kwak EL, Clark JW, Ryan DP, Deshpande V, Dias-Santagata D, Ellisen LW (2012). Frequent mutation of isocitrate dehydrogenase (IDH)1 and IDH2 in cholangiocarcinoma identified through broad-based tumor genotyping. Oncologist.

[26] Kipp BR, Voss JS, Kerr SE, Barr Fritcher EG, Graham RP, Zhang L, Highsmith WE, Zhang J, Roberts LR, Gores GJ, Halling KC (2012). Isocitrate dehydrogenase 1 and 2 mutations in cholangiocarcinoma. Hum Pathol.

[27] Wang P, Dong Q, Zhang C, Kuan PF, Liu Y, Jeck WR, Andersen JB, Jiang W, Savich GL, Tan TX, Auman JT, Hoskins JM, Misher AD, Moser CD, Yourstone SM, Kim JW (2013). Mutations in isocitrate dehydrogenase 1 and 2 occur frequently in intrahepatic cholangiocarcinomas and share hypermethylation targets with glioblastomas. Oncogene.

[28] Arai Y, Totoki Y, Hosoda F, Shirota T, Hama N, Nakamura H, Ojima H, Furuta K, Shimada K, Okusaka T, Kosuge T, Shibata T (2014). Fibroblast growth factor receptor 2 tyrosine kinase fusions define a unique molecular subtype of cholangiocarcinoma. Hepatology.

[29] Graham RP, Barr Fritcher EG, Pestova E, Schulz J, Sitailo LA, Vasmatzis G, Murphy SJ, McWilliams RR, Hart SN, Halling KC, Roberts LR, Gores GJ, Couch FJ, Zhang L, Borad MJ, Kipp BR (2014). Fibroblast growth factor receptor 2 translocations in intrahepatic cholangiocarcinoma. Hum Pathol.

[30] Borad MJ, Champion MD, Egan JB, Liang WS, Fonseca R, Bryce AH, McCullough AE, Barrett MT, Hunt K, Patel MD, Young SW, Collins JM, Silva AC, Condjella RM, Block M, McWilliams RR (2014). Integrated genomic characterization reveals novel, therapeutically relevant drug targets in FGFR and EGFR pathways in sporadic intrahepatic cholangiocarcinoma. PLoS Genet.

[31] Sia D, Losic B, Moeini A, Cabellos L, Hao K, Revill K, Bonal D, Miltiadous O, Zhang Z, Hoshida Y, Cornella H, Castillo-Martin M, Pinyol R, Kasai Y, Roayaie S, Thung SN (2015). Massive parallel sequencing uncovers actionable FGFR2-PPHLN1 fusion and ARAF mutations in intrahepatic cholangiocarcinoma. Nat Commun.

[32] Gu TL, Deng X, Huang F, Tucker M, Crosby K, Rimkunas V, Wang Y, Deng G, Zhu L, Tan Z, Hu Y, Wu C, Nardone J, MacNeill J, Ren J, Reeves C (2011). Survey of tyrosine kinase signaling reveals ROS kinase fusions in human cholangiocarcinoma. PLoS One.

[33] Wiggers JK, Ruys AT, Groot Koerkamp B, Beuers U, ten Kate FJ, van Gulik TM (2014). Differences in immunohistochemical biomarkers between intra- and extrahepatic cholangiocarcinoma: a systematic review and meta-analysis. J Gastroenterol Hepatol.

[34] Kiguchi K, Carbajal S, Chan K, Beltran L, Ruffino L, Shen J, Matsumoto T, Yoshimi N, DiGiovanni J (2001). Constitutive expression of ErbB-2 in gallbladder epithelium results in development of adenocarcinoma. Cancer Res.

[35] Lee J, Park SH, Chang HM, Kim JS, Choi HJ, Lee MA, Jang JS, Jeung HC, Kang JH, Lee HW, Shin DB, Kang HJ, Sun JM, Park JO, Park YS, Kang WK (2012). Gemcitabine and oxaliplatin with or without erlotinib in advanced biliary-tract cancer: a multicentre, open-label, randomised, phase 3 study. Lancet Oncol.

[36] Malka D, Cervera P, Foulon S, Trarbach T, de la Fouchardiere C, Boucher E, Fartoux L, Faivre S, Blanc JF, Viret F, Assenat E, Seufferlein T, Herrmann T, Grenier J, Hammel P, Dollinger M (2014). Gemcitabine and oxaliplatin with or without cetuximab in advanced biliary-tract cancer (BINGO): a randomised, open-label, non-comparative phase 2 trial. Lancet Oncol.

[37] Chen JS, Hsu C, Chiang NJ, Tsai CS, Tsou HH, Huang SF, Bai LY, Chang IC, Shiah HS, Ho CL, Yen CJ, Lee KD, Chiu CF, Rau KM, Yu MS, Yang Y (2015). A KRAS mutation status-stratified randomized phase II trial of gemcitabine and oxaliplatin alone or in combination with cetuximab in advanced biliary tract cancer. Ann Oncol.

[38] Arndt Vogel SK, Weichert W, Bitzer M, Block A, Riess H, Schulze-Bergkamen H, Moehler MH, Merx KE, Endris V, Schnoy E, Siveke JT, Miehl P, Walkdschmidt D (2015). Panitumumab in combination with gemcitabine/cisplatin (GemCis) for patients with advanced kRAS WT biliary tract cancer: A randomized phase II trial of the Arbeitsgemeinschaft Internistische Onkologie (AIO). J Clin Oncol.

[39] Zhu AX, Meyerhardt JA, Blaszkowsky LS, Kambadakone AR, Muzikansky A, Zheng H, Clark JW, Abrams TA, Chan JA, Enzinger PC, Bhargava P, Kwak EL, Allen JN, Jain SR, Stuart K, Horgan K (2010). Efficacy and safety of gemcitabine, oxaliplatin, and bevacizumab in advanced biliary-tract cancers and correlation of changes in 18-fluorodeoxyglucose PET with clinical outcome: a phase 2 study. Lancet Oncol.

[40] Lubner SJ, Mahoney MR, Kolesar JL, Loconte NK, Kim GP, Pitot HC, Philip PA, Picus J, Yong WP, Horvath L, Van Hazel G, Erlichman CE, Holen KD (2010). Report of a multicenter phase II trial testing a combination of biweekly bevacizumab and daily erlotinib in patients with unresectable biliary cancer: a phase II Consortium study. J Clin Oncol.

[41] Iyer RV, Iyer AG, Ma WW, Malhotra U, Iancu D, Grande C, Bekaii-Saab TS (2015). Gemcitabine (G), capecitabine (C) and bevacizumab (BV) in patients with advanced biliary cancers (ABC): final results of a multicenter phase II study. J Clin Oncol.

[42] El-Khoueiry AB, Rankin C, Siegel AB, Iqbal S, Gong IY, Micetich KC, Kayaleh OR, Lenz HJ, Blanke CD (2014). S0941: a phase 2 SWOG study of sorafenib and erlotinib in patients with advanced gallbladder carcinoma or cholangiocarcinoma. Br J Cancer.

[43] El-Khoueiry AB, Rankin CJ, Ben-Josef E, Lenz HJ, Gold PJ, Hamilton RD, Govindarajan R, Eng C, Blanke CD (2012). SWOG 0514: a phase II study of sorafenib in patients with unresectable or metastatic gallbladder carcinoma and cholangiocarcinoma. Invest New Drugs.

[44] Moehler M, Maderer A, Schimanski C, Kanzler S, Denzer U, Kolligs FT, Ebert MP, Distelrath A, Geissler M, Trojan J, Schutz M, Berie L, Sauvigny C, Lammert F, Lohse A, Dollinger MM (2014). Gemcitabine plus sorafenib versus gemcitabine alone in advanced biliary tract cancer: a double-blind placebo-controlled multicentre phase II AIO study with biomarker and serum programme. Eur J Cancer.

[45] Bengala C, Bertolini F, Malavasi N, Boni C, Aitini E, Dealis C, Zironi S, Depenni R, Fontana A, Del Giovane C, Luppi G, Conte P (2010). Sorafenib in patients with advanced biliary tract carcinoma: a phase II trial. Br J Cancer.

[46] Lee JK, Capanu M, O'Reilly EM, Ma J, Chou JF, Shia J, Katz SS, Gansukh B, Reidy-Lagunes D, Segal NH, Yu KH, Chung KY, Saltz LB, Abou-Alfa GK (2013). A phase II study of gemcitabine and cisplatin plus sorafenib in patients with advanced biliary adenocarcinomas. Br J Cancer.

[47] Yi JH, Thongprasert S, Lee J, Doval DC, Park SH, Park JO, Park YS, Kang WK, Lim HY (2012). A phase II study of sunitinib as a second-line treatment in advanced biliary tract carcinoma: a multicentre, multinational study. Eur J Cancer.

[48] Santoro A, Gebbia V, Pressiani T, Testa A, Personeni N, Arrivas Bajardi E, Foa P, Buonadonna A, Bencardino K, Barone C, Ferrari D, Zaniboni A, Tronconi MC, Carteni G, Milella M, Comandone A (2015). A randomized, multicenter, phase II study of vandetanib monotherapy versus vandetanib in combination with gemcitabine versus gemcitabine plus placebo in subjects with advanced biliary tract cancer: the VanGogh study. Ann Oncol.

[49] Valle JW, Wasan H, Lopes A, Backen AC, Palmer DH, Morris K, Duggan M, Cunningham D, Anthoney DA, Corrie P, Madhusudan S, Maraveyas A, Ross PJ, Waters JS, Steward WP, Rees C (2015). Cediranib or placebo in combination with cisplatin and gemcitabine chemotherapy for patients with advanced biliary tract cancer (ABC-03): a randomised phase 2 trial. Lancet Oncol.

[50] Gherardi E, Birchmeier W, Birchmeier C, Vande Woude G (2012). Targeting MET in cancer: rationale and progress. Nat Rev Cancer.

[51] Marquardt JU, Andersen JB (2012). Next-generation sequencing: application in liver cancer-past, present and future?. Biology (Basel).

[52] Pant S, Saleh M, Bendell J, Infante JR, Jones S, Kurkjian CD, Moore KM, Kazakin J, Abbadessa G, Wang Y, Chen Y, Schwartz B, Camacho LH (2014). A phase I dose escalation study of oral c-MET inhibitor tivantinib (ARQ 197) in combination with gemcitabine in patients with solid tumors. Ann Oncol.

[53] Lipika Goyal MBY, Abrams TA, Kwak EL, Cleary JM, Knowles M, Regan E, Gisondi A, Sheehan S, Zheng H, Zhu AX (2015). A phase II trial of cabozantinib (XL-184) in patients with advanced cholangiocarcinoma. J Clin Oncol.

[54] Goyal L, Govindan A, Sheth RA, Nardi V, Blaszkowsky LS, Faris JE, Clark JW, Ryan DP, Kwak EL, Allen JN, Murphy JE, Saha SK, Hong TS, Wo JY, Ferrone CR, Tanabe KK (2015). Prognosis and Clinicopathologic Features of Patients With Advanced Stage Isocitrate Dehydrogenase (IDH) Mutant and IDH Wild-Type Intrahepatic Cholangiocarcinoma. Oncologist.

[55] Grassian AR, Pagliarini R, Chiang DY (2014). Mutations of isocitrate dehydrogenase 1 and 2 in intrahepatic cholangiocarcinoma. Curr Opin Gastroenterol.

[56] Rohle D, Popovici-Muller J, Palaskas N, Turcan S, Grommes C, Campos C, Tsoi J, Clark O, Oldrini B, Komisopoulou E, Kunii K, Pedraza A, Schalm S, Silverman L, Miller A, Wang F (2013). An inhibitor of mutant IDH1 delays growth and promotes differentiation of glioma cells. Science.

[57] Wang F, Travins J, DeLaBarre B, Penard-Lacronique V, Schalm S, Hansen E, Straley K, Kernytsky A, Liu W, Gliser C, Yang H, Gross S, Artin E, Saada V, Mylonas E, Quivoron C (2013). Targeted inhibition of mutant IDH2 in leukemia cells induces cellular differentiation. Science.

[58] Tiong KH, Mah LY, Leong CO (2013). Functional roles of fibroblast growth factor receptors (FGFRs) signaling in human cancers. Apoptosis.

[59] Wu YM, Su F, Kalyana-Sundaram S, Khazanov N, Ateeq B, Cao X, Lonigro RJ, Vats P, Wang R, Lin SF, Cheng AJ, Kunju LP, Siddiqui J, Tomlins SA, Wyngaard P, Sadis S (2013). Identification of targetable FGFR gene fusions in diverse cancers. Cancer Discov.

[60] Nakamura H, Arai Y, Totoki Y, Shirota T, Elzawahry A, Kato M, Hama N, Hosoda F, Urushidate T, Ohashi S, Hiraoka N, Ojima H, Shimada K, Okusaka T, Kosuge T, Miyagawa S (2015). Genomic spectra of biliary tract cancer. Nat Genet.

[61] Takahashi R, Yoshitomi M, Yutani S, Shirahama T, Noguchi M, Yamada A, Itoh K, Sasada T (2013). Current status of immunotherapy for the treatment of biliary tract cancer. Hum Vaccin Immunother.

[62] Oshikiri T, Miyamoto M, Shichinohe T, Suzuoki M, Hiraoka K, Nakakubo Y, Shinohara T, Itoh T, Kondo S, Katoh H (2003). Prognostic value of intratumoral CD8+ T lymphocyte in extrahepatic bile duct carcinoma as essential immune response. J Surg Oncol.

[63] Lipson EJ, Drake CG (2011). Ipilimumab: an anti-CTLA-4 antibody for metastatic melanoma. Clin Cancer Res.

[64] Sullivan RJ, Flaherty KT (2015). Pembrolizumab for Treatment of Patients with Advanced or Unresectable Melanoma. Clin Cancer Res.

[65] Koido S, Kan S, Yoshida K, Yoshizaki S, Takakura K, Namiki Y, Tsukinaga S, Odahara S, Kajihara M, Okamoto M, Ito M, Yusa S, Gong J, Sugiyama H, Ohkusa T, Homma S (2014). Immunogenic modulation of cholangiocarcinoma cells by chemoimmunotherapy. Anticancer Res.

[66] Le DT, Uram JN, Wang H, Bartlett BR, Kemberling H, Eyring AD, Skora AD, Luber BS, Azad NS, Laheru D, Biedrzycki B, Donehower RC, Zaheer A, Fisher GA, Crocenzi TS, Lee JJ (2015). PD-1 Blockade in Tumors with Mismatch-Repair Deficiency. N Engl J Med.

[67] Saborowski A, Saborowski M, Davare MA, Druker BJ, Klimstra DS, Lowe SW (2013). Mouse model of intrahepatic cholangiocarcinoma validates FIG-ROS as a potent fusion oncogene and therapeutic target. Proc Natl Acad Sci U S A.

[68] Solomon BJ, Mok T, Kim DW, Wu YL, Nakagawa K, Mekhail T, Felip E, Cappuzzo F, Paolini J, Usari T, Iyer S, Reisman A, Wilner KD, Tursi J, Blackhall F, Investigators P (2014). First-line crizotinib versus chemotherapy in ALK-positive lung cancer. N Engl J Med.

[69] Andersen JB, Spee B, Blechacz BR, Avital I, Komuta M, Barbour A, Conner EA, Gillen MC, Roskams T, Roberts LR, Factor VM, Thorgeirsson SS (2012). Genomic and genetic characterization of cholangiocarcinoma identifies therapeutic targets for tyrosine kinase inhibitors. Gastroenterology.

[70] Voss JS, Holtegaard LM, Kerr SE, Fritcher EG, Roberts LR, Gores GJ, Zhang J, Highsmith WE, Halling KC, Kipp BR (2013). Molecular profiling of cholangiocarcinoma shows potential for targeted therapy treatment decisions. Hum Pathol.

[71] Xu RF, Sun JP, Zhang SR, Zhu GS, Li LB, Liao YL, Xie JM, Liao WJ (2011). KRAS and PIK3CA but not BRAF genes are frequently mutated in Chinese cholangiocarcinoma patients. Biomed Pharmacother.

[72] Lee D, Do IG, Choi K, Sung CO, Jang KT, Choi D, Heo JS, Choi SH, Kim J, Park JY, Cha HJ, Joh JW, Choi KY, Kim DS (2012). The expression of phospho-AKT1 and phospho-MTOR is associated with a favorable prognosis independent of PTEN expression in intrahepatic cholangiocarcinomas. Mod Pathol.

[73] Ewald F, Norz D, Grottke A, Hofmann BT, Nashan B, Jucker M (2014). Dual Inhibition of PI3K-AKT-mTOR- and RAF-MEK-ERK-signaling is synergistic in cholangiocarcinoma and reverses acquired resistance to MEK-inhibitors. Invest New Drugs.

[74] Ewald F, Grabinski N, Grottke A, Windhorst S, Norz D, Carstensen L, Staufer K, Hofmann BT, Diehl F, David K, Schumacher U, Nashan B, Jucker M (2013). Combined targeting of AKT and mTOR using MK-2206 and RAD001 is synergistic in the treatment of cholangiocarcinoma. Int J Cancer.

[75] McRee AJ, Sanoff HK, Carlson C, Ivanova A, O'Neil BH (2015). A phase I trial of mFOLFOX6 combined with the oral PI3K inhibitor BKM120 in patients with advanced refractory solid tumors. Invest New Drugs.

[76] Costello BA, Borad MJ, Qi Y, Kim GP, Northfelt DW, Erlichman C, Alberts SR (2014). Phase I trial of everolimus, gemcitabine and cisplatin in patients with solid tumors. Invest New Drugs.

[77] O'Neill E, Kolch W (2004). Conferring specificity on the ubiquitous Raf/MEK signalling pathway. Br J Cancer.

[78] Chen TC, Jan YY, Yeh TS (2012). K-ras mutation is strongly associated with perineural invasion and represents an independent prognostic factor of intrahepatic cholangiocarcinoma after hepatectomy. Ann Surg Oncol.

[79] Robertson S, Hyder O, Dodson R, Nayar SK, Poling J, Beierl K, Eshleman JR, Lin MT, Pawlik TM, Anders RA (2013). The frequency of KRAS and BRAF mutations in intrahepatic cholangiocarcinomas and their correlation with clinical outcome. Hum Pathol.

[80] Bekaii-Saab T, Phelps MA, Li X, Saji M, Goff L, Kauh JS, O'Neil BH, Balsom S, Balint C, Liersemann R, Vasko VV, Bloomston M, Marsh W, Doyle LA, Ellison G, Grever M (2011). Multi-institutional phase II study of selumetinib in patients with metastatic biliary cancers. J Clin Oncol.

[81] Bridgewater J, Lopes A, Beare S, Duggan M, Lee D, Ricamara M, McEntee D, Sukumaran A, Wasan H, Valle JW (2016). A phase 1b study of Selumetinib in combination with Cisplatin and Gemcitabine in advanced or metastatic biliary tract cancer: the ABC-04 study. BMC Cancer.

[82] Tannapfel A, Sommerer F, Benicke M, Katalinic A, Uhlmann D, Witzigmann H, Hauss J, Wittekind C (2003). Mutations of the BRAF gene in cholangiocarcinoma but not in hepatocellular carcinoma. Gut.

[83] Hyman DM, Puzanov I, Subbiah V, Faris JE, Chau I, Blay JY, Wolf J, Raje NS, Diamond EL, Hollebecque A, Gervais R, Elez-Fernandez ME, Italiano A, Hofheinz RD, Hidalgo M, Chan E (2015). Vemurafenib in Multiple Nonmelanoma Cancers with BRAF V600 Mutations. N Engl J Med.

[84] Shen DY, Zhang W, Zeng X, Liu CQ (2013). Inhibition of Wnt/beta-catenin signaling downregulates P-glycoprotein and reverses multi-drug resistance of cholangiocarcinoma. Cancer Sci.

[85] Denlinger CS, Meropol NJ, Li T, Lewis NL, Engstrom PF, Weiner LM, Cheng JD, Alpaugh RK, Cooper H, Wright JJ, Cohen SJ (2014). A phase II trial of the proteasome inhibitor bortezomib in patients with advanced biliary tract cancers. Clin Colorectal Cancer.

[86] Sia D, Tovar V, Moeini A, Llovet JM (2013). Intrahepatic cholangiocarcinoma: pathogenesis and rationale for molecular therapies. Oncogene.

[87] Sia D, Hoshida Y, Villanueva A, Roayaie S, Ferrer J, Tabak B, Peix J, Sole M, Tovar V, Alsinet C, Cornella H, Klotzle B, Fan JB, Cotsoglou C, Thung SN, Fuster J (2013). Integrative molecular analysis of intrahepatic cholangiocarcinoma reveals 2 classes that have different outcomes. Gastroenterology.

[88] Hedvat M, Huszar D, Herrmann A, Gozgit JM, Schroeder A, Sheehy A, Buettner R, Proia D, Kowolik CM, Xin H, Armstrong B, Bebernitz G, Weng S, Wang L, Ye M, McEachern K (2009). The JAK2 inhibitor AZD1480 potently blocks Stat3 signaling and oncogenesis in solid tumors. Cancer Cell.

[89] Zender S, Nickeleit I, Wuestefeld T, Sorensen I, Dauch D, Bozko P, El-Khatib M, Geffers R, Bektas H, Manns MP, Gossler A, Wilkens L, Plentz R, Zender L, Malek NP (2013). A critical role for notch signaling in the formation of cholangiocellular carcinomas. Cancer Cell.

[90] El Khatib M, Bozko P, Palagani V, Malek NP, Wilkens L, Plentz RR (2013). Activation of Notch signaling is required for cholangiocarcinoma progression and is enhanced by inactivation of p53 *in vivo*. PLoS One.

[91] Sekiya S, Suzuki A (2012). Intrahepatic cholangiocarcinoma can arise from Notch-mediated conversion of hepatocytes. J Clin Invest.

[92] Wu WR, Zhang R, Shi XD, Zhu MS, Xu LB, Zeng H, Liu C (2014). Notch1 is overexpressed in human intrahepatic cholangiocarcinoma and is associated with its proliferation, invasiveness and sensitivity to 5-fluorouracil *in vivo*. Oncol Rep.

[93] Loilome W, Juntana S, Namwat N, Bhudhisawasdi V, Puapairoj A, Sripa B, Miwa M, Saya H, Riggins GJ, Yongvanit P (2011). PRKAR1A is overexpressed and represents a possible therapeutic target in human cholangiocarcinoma. Int J Cancer.

[94] Reya T, Clevers H (2005). Wnt signalling in stem cells and cancer. Nature.

[95] Boulter L, Guest RV, Kendall TJ, Wilson DH, Wojtacha D, Robson AJ, Ridgway RA, Samuel K, Van Rooijen N, Barry ST, Wigmore SJ, Sansom OJ, Forbes SJ (2015). WNT signaling drives cholangiocarcinoma growth and can be pharmacologically inhibited. J Clin Invest.

[96] Huang GL, Shen DY, Cai CF, Zhang QY, Ren HY, Chen QX (2015). beta-escin reverses multidrug resistance through inhibition of the GSK3beta/beta-catenin pathway in cholangiocarcinoma. World J Gastroenterol.

[97] Philip PA, Mahoney MR, Allmer C, Thomas J, Pitot HC, Kim G, Donehower RC, Fitch T, Picus J, Erlichman C (2006). Phase II study of erlotinib in patients with advanced biliary cancer. J Clin Oncol.

[98] Gruenberger B, Schueller J, Heubrandtner U, Wrba F, Tamandl D, Kaczirek K, Roka R, Freimann-Pircher S, Gruenberger T (2010). Cetuximab, gemcitabine, and oxaliplatin in patients with unresectable advanced or metastatic biliary tract cancer: a phase 2 study. Lancet Oncol.

[99] Paule B, Herelle MO, Rage E, Ducreux M, Adam R, Guettier C, Bralet MP (2007). Cetuximab plus gemcitabine-oxaliplatin (GEMOX) in patients with refractory advanced intrahepatic cholangiocarcinomas. Oncology.

[100] Rubovszky G, Lang I, Ganofszky E, Horvath Z, Juhos E, Nagy T, Szabo E, Szentirmay Z, Budai B, Hitre E (2013). Cetuximab, gemcitabine and capecitabine in patients with inoperable biliary tract cancer: a phase 2 study. Eur J Cancer.

[101] Borbath I, Ceratti A, Verslype C, Demols A, Delaunoit T, Laurent S, Deleporte A, Vergauwe P, Van Maanen A, Sempoux C, Van Cutsem E, Van Laethem JL, Belgian Group of Digestive O (2013). Combination of gemcitabine and cetuximab in patients with advanced cholangiocarcinoma: a phase II study of the Belgian Group of Digestive Oncology. Ann Oncol.

[102] Jensen LH, Lindebjerg J, Ploen J, Hansen TF, Jakobsen A (2012). Phase II marker-driven trial of panitumumab and chemotherapy in KRAS wild-type biliary tract cancer. Ann Oncol.

[103] Hezel AF, Noel MS, Allen JN, Abrams TA, Yurgelun M, Faris JE, Goyal L, Clark JW, Blaszkowsky LS, Murphy JE, Zheng H, Khorana AA, Connolly GC, Hyrien O, Baran A, Herr M (2014). Phase II study of gemcitabine, oxaliplatin in combination with panitumumab in KRAS wild-type unresectable or metastatic biliary tract and gallbladder cancer. Br J Cancer.

[104] Sohal DP, Mykulowycz K, Uehara T, Teitelbaum UR, Damjanov N, Giantonio BJ, Carberry M, Wissel P, Jacobs-Small M, O'Dwyer PJ, Sepulveda A, Sun W (2013). A phase II trial of gemcitabine, irinotecan and panitumumab in advanced cholangiocarcinoma. Ann Oncol.

[105] Ramanathan RK, Belani CP, Singh DA, Tanaka M, Lenz HJ, Yen Y, Kindler HL, Iqbal S, Longmate J, Mack PC, Lurje G, Gandour-Edwards R, Dancey J, Gandara DR (2009). A phase II study of lapatinib in patients with advanced biliary tree and hepatocellular cancer. Cancer Chemother Pharmacol.

